# Factors Associated with Parental Non-Adoption of Infant Male Circumcision for HIV Prevention in Sub-Saharan Africa: A Systematic Review and Thematic Synthesis

**DOI:** 10.1007/s10461-014-0835-7

**Published:** 2014-07-01

**Authors:** Webster Mavhu, Zivai Mupambireyi, Graham Hart, Frances M. Cowan

**Affiliations:** 1 Centre for Sexual Health and HIV/AIDS Research (CeSHHAR) Zimbabwe, 9 Monmouth Road, Avondale, Harare, Zimbabwe; 2 Centre for Sexual Health and HIV Research, Research Department of Infection and Population Health, University College London, London, UK

**Keywords:** Infant male circumcision, Barriers, Qualitative, Interventions, Sub-Saharan Africa

## Abstract

Infant male circumcision (IMC) may be more effective at preventing HIV than adult male circumcision as the procedure is carried out before the individual becomes sexually active. Successful scale-up will depend on identifying and overcoming parental concerns that may act as barriers for IMC. We conducted a systematic review to identify qualitative studies reporting on parental reasons for non-adoption of IMC for HIV prevention in sub-Saharan Africa. Thematic synthesis was subsequently conducted. Five descriptive themes were identified; these were later condensed into two main analytical themes: “poor knowledge” and “social constructs”. While barriers and motivators are to some degree context specific, this review suggests that there are common themes that need to be addressed across the region if uptake of IMC for HIV prevention is to be widely adopted. Study findings are therefore likely to have broad implications for IMC roll out.

**Electronic supplementary material:**

The online version of this article (doi:10.1007/s10461-014-0835-7) contains supplementary material, which is available to authorized users.

## Resumen

Circuncisión infantil (IMC) puede ser más eficaz en la prevención del VIH que MC adultos como el procedimiento se lleva a cabo antes de que el individuo llega a ser sexualmente activo. Escalado exitosa dependerá de identificar y superar las preocupaciones parentales que pueden actuar como barreras para IMC. Se realizó una revisión sistemática para identificar estudios cualitativos informando sobre las razones de los padres para la no adopción de IMC para la prevención del VIH en África subsahariana. Posteriormente se realizó la síntesis temática. Se identificaron cinco temas descriptivos; Éstos fueron posteriormente condensadas en dos temas principales de análisis: “pobre conocimiento” y “construcciones sociales”. Mientras que las barreras y motivadores son poco grado de contexto específico, esta revisión sugiere que existen temas comunes que deben abordarse en toda la región si la absorción de IMC para la prevención del VIH va a ser ampliamente adoptado. Resultados del estudio por lo tanto, son propensos a tener amplias implicaciones para despliegue IMC.

## Introduction

By 2007 three randomized controlled trials (RCTs) conducted in sub-Saharan Africa had conclusively demonstrated efficacy of male circumcision (MC) in reducing the risk of HIV infection in heterosexual men by up to 60 % [1–3]. Longer-term follow up suggests that the protective effect of male circumcision persists [4, 5], and recent findings on population-level impact from South Africa confirm those from the RCTs [6]. Based on RCT results, the World Health Organization (WHO) has recommended rapid scale-up of voluntary medical male circumcision (VMMC) in 14 Eastern and Southern African countries with high HIV prevalence and low rates of male circumcision [7–9]. These countries are Botswana, Ethiopia, Kenya, Lesotho, Malawi, Mozambique, Namibia, Rwanda, South Africa, Swaziland, Uganda, Tanzania, Zambia and Zimbabwe [7–9].

Modelling studies conducted between 2009 and 2011 estimated that circumcising 80 % of males aged 15–49 in the 14 African countries within 5 years and sustaining this coverage rate thereafter, could avert 3.4 million new HIV infections within 15 years, in addition to yielding savings of US$16.5 billion in care and treatment costs [10, 11]. Thus, the 14 VMMC priority countries are striving to scale-up VMMC to a level that could impact the transmission of HIV [12]. In order to ensure that benefits of MC are sustained in the longer-term, WHO also recommends that infant MC (IMC) be implemented alongside adult VMMC [7]. Presuming high uptake, it will then be possible to phase out the “catch up” adult VMMC as infants circumcised now come of age. Pilot implementation of early infant male circumcision is already underway in some of the 14 VMMC priority countries including Botswana, Kenya, Lesotho, Swaziland, Zambia, and Zimbabwe [13–18].

Although its effects on HIV will take longer to realize, infant MC is likely to ultimately be more effective at preventing HIV acquisition than adult MC since infant MC is conducted long before the individual becomes sexually active, thereby negating the risk of sexual acquisition of HIV before male circumcision and the risk associated with sex before complete wound healing after the procedure [19]. Resumption of sex before the recommended post-circumcision abstinence period (6 weeks) is a major issue with adult men [1, 20, 21]. Since actual acceptability of IMC will have a bearing on the procedure’s uptake, roll-out and subsequent impact, it is crucial to identify and address parental concerns that may act as barriers for infant MC for HIV prevention. Identifying parental barriers and specifically working to address them will likely improve uptake and maximise the intervention’s benefits.

This study sought to collate qualitative data which explore parental reasons for non-adoption of infant MC for HIV prevention in sub-Saharan Africa. The main question that the study sought to answer is ‘For what reasons might parents choose not to adopt infant MC for HIV prevention in sub-Saharan Africa?’ Findings will be used to inform the development of a package of approaches for overcoming these parental barriers (that could subsequently be tested for impact). The synthesis is described in line with a recently-developed set of guidelines for reporting synthesis of qualitative studies—‘Enhancing transparency in reporting the synthesis of qualitative research (ENTREQ)’ [22]. ENTREQ consists of 21 items grouped into five main domains: introduction, methods and methodology, literature search and selection, appraisal, and synthesis of findings [22].

In addition to being comparatively new (albeit growing), the practice of synthesising qualitative studies is a subject of on-going debate [23, 24]. Some maintain that it is not valid to take qualitative findings from a specific context, time and group and generalise beyond that setting [25, 26]. However, a strong case has been made for qualitative synthesis to be valued as it brings together qualitative evidence from a range of settings for a wider audience, identifies research gaps and provides evidence for health-care and policy [22–24, 26–29]. The described synthesis was conducted in the hope that it would contribute to the design of interventions to tackle parental barriers that need to be addressed in order to facilitate infant male circumcision adoption in sub-Saharan Africa in general and Zimbabwe, specifically.

## Methods

### Inclusion Criteria

Studies were selected for review if they were published in peer-reviewed journals; reported qualitative data on barriers to infant MC for HIV prevention entirely or in combination with quantitative ones, and were conducted in sub-Saharan Africa. Studies were excluded if they reported only quantitative data, were conducted outside sub-Saharan Africa, and focused only on adult MC. Abstracts for conference proceedings were excluded because abstracts for qualitative studies seldom provide sufficiently detailed methods and results, making it difficult to judge their suitability for synthesis [24].

### Search Strategy

To develop the search strategy, we first split the research question into four components: (a) male or infant circumcision, (b) HIV prevention, (c) acceptability, and (d) qualitative research. Synonyms for the four components were identified through reading relevant literature. Additional synonyms were identified through a review of pilot search results. The search was first conducted using Medline Medical Subject Headings (MeSH) terms and text searches. Thereafter, two searches were conducted in Embase and CINAHL Plus using key terms and text words (plus thesaurus) relevant to each database. In addition to allowing for adjacency for words in a phrase, word endings were truncated to ensure inclusiveness of text searches. The Boolean operator “OR” was used to identify all papers related to each component after which search returns of all four components were combined using the operator “AND”. The final search in the three databases was run and closed on 22 January 2013. Identified papers from each of the databases were imported into an Endnote reference management software file (library). Duplicates were identified and removed.

### Selection of Eligible Papers

Titles and abstracts were used to screen papers for relevance to the systematic review. If it was clear that a paper was ineligible based on the title and/or abstract review, it was dropped. Where the title and/or abstract were insufficient to make a determination, the full paper was downloaded and read. If the paper was deemed ineligible, it was excluded and reasons for exclusion were documented. Reference lists for all eligible papers were scrutinised for any additional relevant papers.

### Quality Assessment

Assessing quality of qualitative research is not only challenging but also contentious [22, 23]. There is little consensus on how quality should be assessed as well as whether it can or should be assessed at all [23, 29]. We assessed selected studies using an adaptation of previously-derived quality criteria for assessing validity of qualitative research [26, 30]. Two experienced qualitative researchers (WM and ZM) first conducted this process independently and then jointly. The adapted criteria covered three main issues: *reporting* of study methods, *reporting* of study findings, and *interpretation* of study findings (Table 1).

**Table 1:** Criteria used to assess quality of studies

*Items on reporting of study methods*
1. Paper reports findings from qualitative methodology
2. Clear statement on aims/objectives
3. Sampling strategy explained and appropriate
4. Data collection methods mentioned/described and appropriate
5. Mention of ethical considerations
6. Theoretical approach mentioned/described
7. Analysis adequately described
8. Analysis done by more than one person to minimise subjectivity
*Items on reporting of study findings*
9. Results can be linked back to study objectives
10. Sufficient data presented to support the results (including quotes)
*Items on interpretation of study findings*
11. Discussion and conclusions adequately supported by the data

### Synthesis

Electronic copies of the 10 studies were directly imported into NVivo 10 (QSR International, Melbourne, Australia), a qualitative data storage and retrieval program. The thematic synthesis of findings was done in three previously-validated and recommended stages: line-by-line coding of study findings, developing descriptive themes and generating analytical themes [23, 28]. *Stage one: line-by-line coding of study findings*. Two researchers independently conducted line-by-line coding of qualitative findings presented in the selected studies. During this stage of the synthesis, the two researchers also examined each other’s codes to check consistency of interpretation and to see whether additional levels of coding were needed [23].

*Stage two: developing descriptive themes*. The two reviewers looked for similarities and differences between the codes in order to group them [23]. New codes were created to capture the meaning of groups of initial codes through an iterative, inductive process. This process resulted in 5 descriptive themes, 15 sub-themes and 8 sub sub-themes. Due to the overlapping nature of the 3 stages of thematic synthesis, some codes were adopted as themes/sub-themes in their original form.

*Stage three: generating analytical themes*. Stage three involved condensing the descriptive themes into analytical ones [23]. Reviewers analysed the barriers to infant MC suggested by the descriptive themes, sub-themes, sub sub-themes, condensed these, and then considered their implications for possible interventions. Each reviewer first did this independently and then together. This process resulted in two main analytical themes and several recommendations for possible interventions.

## Results

**Fig. 1:**
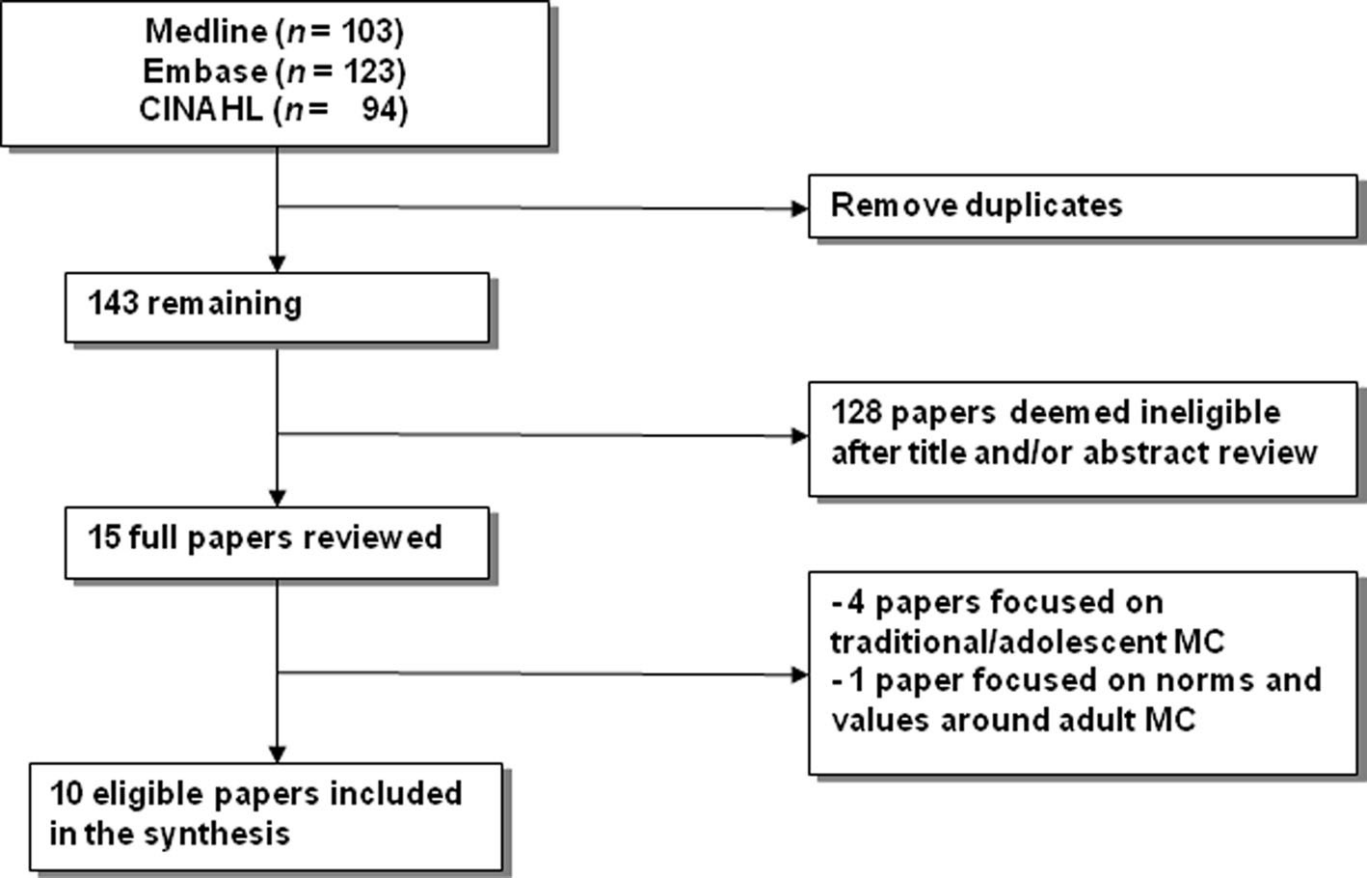
Selection of eligible papers

Our search conducted to 22 January 2013 resulted in 320 hits, which included 177 duplicate articles. After removing duplicates, a further 128 were excluded based on the title and/or abstract review (see Fig. 1). Studies were excluded for at least one of the following reasons: [1] they reported only quantitative data, [2] they were conducted outside sub-Saharan Africa, and [3] they focused only on adult MC. The full article was read for the remaining 15 articles. Of these, five papers were excluded from the analysis because they failed to meet the inclusion criteria; four [31–34] because they focused on traditional adolescent MC and one [35] because it reported on norms and values around adult MC (see Fig. 1 for details of the selection process). Ten papers met the inclusion criteria and were assessed for quality and assigned a quality score ranging from poor to fair/good (see Table 2).

**Table 2:** Characteristics of included studies

Date	Country	Authors (ref)	Time of study	Study population	Setting	Data collection methods	Quality rating
2002	Kenya	Bailey et al. [37]	1998	16–80 year-old men and women	Rural and urban	Focus groups and interviews	Good
2003	South Africa	Rain-Taljaard et al. [43]	1999–2000	13–59 year-old men and women	Peri-urban	Focus groups and interviews	Fair
2006	Malawi	Ngalande et al. [42]	2003	16–80 year-old men and women	Rural and urban	Focus groups	Good
2007	Zambia	Lukobo and Bailey [38]	2003	18–67 year-old men	Rural and urban	Focus groups	Fair
2011	Tanzania	Mwanga et al. [41]	2008-2009	40–59 year-old men and women	Rural and urban	Interviews	Good
2011	Uganda	Albert et al. [36]	2008	16–80 year-old men and women	Rural and urban	Focus groups	Fair
2012	Tanzania	Tarimo et al. [44]	2009	24 male and 10 female police officers	Urban	Interviews	Good
2012	South Africa	Milford et al. [40]	2008	16 females and 4 males	Rural and urban	Interviews	Good
2012	Zambia	Waters et al. [45]	2009–2010	18–74 year-old men and women	Urban	Focus groups	Good
2012	Zimbabwe	Mavhu et al. [39]	2010	16–80 year-old men and women	Rural and urban	Focus groups and interviews	Good

### Characteristics of Included Studies

The 10 studies [36–45] included in the synthesis were conducted in 7 countries [Kenya, Malawi, South Africa (×2), Tanzania (×2), Uganda, Zambia (×2), and Zimbabwe]. The earliest paper [37] was published in 2002 and the most recent [39] in 2012. Two studies [39, 45] conducted between 2009 and 2010 specifically explored acceptability of infant MC. Seven of the 10 studies (70 %) were perceived to be of good quality and three (30 %) of fair quality (see Table 2). Overall, studies rated as fair either did not report how data analysis was done and/or did not include participants’ verbatim quotes to substantiate findings. Two of the three papers rated as fair [36, 43] reported mixed methods research. In the first case [36], qualitative research was ancillary to household and provider surveys. In the second [43], focus group discussions (FGDs) and in-depth interviews were conducted alongside two cross-sectional studies.

### Results of Synthesis: Line-By-Line Coding

Line-by-line coding resulted in 24 codes (Table 3). The codes are listed in order of the two researchers’ own perceptions of their relative weight with regards to influencing non-adoption of infant MC (with 1 being perceived as most significant) and not by the number of times each code appears in the studies. Although some of the codes are closely related, they have certain nuances within them and as such, we felt it was justifiable to treat them separately. However, the codes were subsequently grouped into themes, sub-themes and sub sub-themes as illustrated in Table 4.

**Table 3:** Codes identified from the studies

Code	Frequency
1. Fear of death	5
2. Fear of negative outcome	4
3. Fear of HIV infection	1
4. Fear of excessive bleeding	6
5. Fear of excessive pain	5
6. Fear of infection	2
7. Don’t understand rationale	1
8. Scepticism about preventative benefits	1
9. Lack of confidence in procedure safety	2
10. Lack of confidence in medical personnel	2
11. Not understanding advantages of infant MC over later in childhood	2
12. Concerns about cost	5
13. Suspicion about/of program	1
14. Unfamiliarity with procedure, father uncircumcised	2
15. Fear of loss of penile sensitivity	2
16. Fear of loss of sexual desire	2
17. Non-MC a major distinguishing feature	1
18. Preserving tradition	4
19. Fear of rejection/derision/ostracism	3
20. Associated religious connotations	2
21. Fear of witchcraft	2
22. Fear of excessive sexual desire (womanizing)	1
23. Fear of risky sexual activity/behaviour later	2
24. Respect for child autonomy	2

**Table 4:** Themes—parental reasons for not adopting infant male circumcision for HIV prevention

Theme	
*Lack of information*	
Rationale	“Fathers themselves have not gone for circumcision and so they do not believe it is important for their children” ([45], p 17)
Preventative benefits	“I am still not convinced that circumcision reduces the spread of HIV” ([45], p17)
Advantages (over MC later in life)	“It [MC] should be done when they [babies] are about six months old” ([39], p 2)
*Fear of harm*	
Immediate harm	
Death	“They [providers] might kill my child, like the case in Kafue of the child that died after an MC operation” ([45], p 17)
HIV infection	“The most commonly expressed reasons not to circumcise were fear of infection, including HIV…” ([42], p 381)
Excessive bleeding	“The danger of excessive bleeding is of particular concern for mothers considering circumcision for young children…Some said that infants and small boys *simply do not have sufficient blood to spare*” (our emphasis) ([37], p 31)
Excessive pain	“Many were against circumcising babies because of excessive pain during and after the procedure” ([38], p 474)
Infection	“Although you may be told the instruments were boiled [sterilized], you may find out that they are not clean… Perhaps, they [instruments] have been there for a long time and bacteria are there; then without knowing all that, a person is circumcised using those instruments” ([44], p 7)
Future harm	
Physical	
Decreased penile sensitivity	“Additional barriers to male circumcision mentioned by a few participants included some loss of penile sensitivity…” ([37], p 31)
Decreased sexual desire	“Additional barriers to male circumcision mentioned by a few participants included some loss of … and sexual desire…” ([37], p 31)
Behavioural	
Increased sexual desire	“Additional barriers to male circumcision mentioned by a few participants included …excessive sexual desire and a tendency to womanize” ([37], p 31)
Risk compensation	“…FGDs also raised a number of concerns and challenges to MC promotion. These included concerns …that it would encourage their children to engage in risky sexual activity” ([36], p 1582)
Social	
Ostracism	“Some of these individuals were concerned that boys who were circumcised might be ostracized from their church communities” ([42], p 382)
Derision	“Many said that Luo boys and men would want to avoid being called *rayuom*, a derisive DhoLuo term for the circumcised and for those born with reduced foreskin” ([37], p 30)
Rejection	“A few suggested that Luo women might reject a Luo man as potential sex or marriage partner if he is circumcised” ([37], p 30)
Religious connotations	“Among those from non circumcising tribes, several participants described circumcision as cultural practice associated with Muslims…” ([45], p 16)
*Culture/traditional beliefs*	
Maintaining tradition	“‘Infants would need to be nursed by their mothers [after circumcision) We don’t want mothers to know what we do’” ([39], p 3)
Non-MC a distinguishing feature	“Until recently, extraction of the lower middle six teeth was an identifying feature for Luo as they passed into adulthood, but now that tooth extraction has been largely abandoned, participants see lack of male circumcision as the most significant component of Luo identity aside from language. A few were concerned that, if Luo started circumcising, little would be left to distinguish them from others” ([37], p 30)
Witchcraft	“I wanted to have my son circumcised, but my husband refused. He said it was a practice connected to witchcraft” ([45], p 17)
*Concerns about cost*	“Cost of the procedure was expressed by many groups as a significant barrier to circumcision for themselves or their sons” ([37], p 31)
*Respect for child autonomy*	“In the past elders were doing tattoos (Ndembo) on our bodies, and now I understand that these were not good, which is why I wouldn’t want to decide on circumcision for someone else. We may think we are doing the right thing when in fact our children may disagree when they grow up” ([45], p 17)

### Results of Synthesis: Descriptive Themes

In summary, barriers to non-adoption of infant male circumcision for HIV prevention in sub-Saharan Africa include a lack of information. An additional recurrent theme is fear of harm—both immediate (infant death, HIV infection, excessive bleeding and pain, infection) and future (decreased penile sensitivity and sexual desire, increased sexual desire, risk compensation, ostracism, derision and rejection). Cultural and traditional beliefs also seem to be significant barriers. Concerns about cost emerged as a significant barrier especially within the context of competing interests. Surprisingly, the need to respect a child’s autonomy featured (albeit in only one setting) as a barrier despite the fact that in most African settings, children seldom feel able to make independent decisions (Table 4). All of these factors act as barriers to adoption of infant MC for HIV prevention by the male infant’s parents.

### Results of Synthesis: Analytical Themes

Poor knowledge of infant MC and its potential benefits is a barrier to intervention uptake. Parents neither understand the rationale behind infant MC nor what the procedure involves. Some doubt (infant) MC’s effectiveness in protecting males against HIV. Social constructs—taken here to mean ideas created and sustained by an individual or group—are a source of barriers to infant MC. These include societal myths and misconceptions. Some of the myths act as barriers to MC for HIV prevention because they are associated with threats to a specific social construct—masculinity (e.g. MC decreases sexual desire or pleasure) [46].

## Discussion

This paper presents a thematic analysis of systematically identified qualitative studies from sub-Saharan Africa that reported barriers to infant male circumcision for HIV prevention. Five major themes were identified: lack of information, fear of harm, cultural/traditional beliefs, concerns about cost and need to respect a child’s autonomy.

Several studies identified poor knowledge as a barrier to infant MC for HIV prevention, suggesting that campaigns designed to create demand for the intervention need to provide parents with accurate information about the efficacy of MC in preventing HIV (as well as its other health benefits). Misconceptions about how the procedure is conducted and the risks associated with it were commonly cited; information, education, and communication (IEC) materials will need to provide understandable and accurate information to explain the procedure and that when conducted by appropriately trained and experienced personnel, IMC is safe, does not require sutures and is usually characterised by minimal bleeding [15, 18]. Additionally, IEC materials need to also explain issues around pain management and infection control (and that if an infection occurs, it usually involves just the skin and can be easily treated) [15, 18]. Concerns around the possibility that infant MC may itself be a source of HIV infection need to be specifically addressed. IEC materials should also highlight the several advantages of circumcising males during infancy as opposed to later in life.

Concerns around both the monetary and opportunity cost of the procedure were common. Infant MC programmes will need to ensure that the cost of the procedure is minimised, for example, by providing the procedure and follow up without charge to offset at least some of the primary and opportunity costs. This approach has been adopted by some adult VMMC priority countries (Kenya, Malawi, Rwanda, Swaziland, Tanzania and Zimbabwe) [46]. However, social marketing theory suggests that people generally do not value something that they get for free [47]. Moreover, in one of the studies included in this review, some participants questioned the motive of providing free male circumcision, particularly when funded by foreign donors [39]. In a separate study, participants recommended low priced male circumcision to boost the community’s satisfaction with the quality of the procedure [42, 48]. Asking infant MC clients to pay a nominal procedure fee might help to increase the value people attach to IMC.

Social constructs, a potential barrier to infant MC, need to be tackled. In addition to tackling masculinity, interventions to promote infant MC for HIV prevention need to dispel MC-related myths and misconceptions. Since fear of various forms of social maltreatment, including ostracism by church members were reported, initiatives to provide information about infant MC for HIV prevention could be incorporated into faith-based HIV prevention interventions; these have been successfully used in sub-Saharan Africa [49, 50]. Non-circumcising communities view MC in general and infant MC, specifically, as a form of conversion to the ‘other’ and a loss of cultural identity. Where circumcision is seen as a “backward” practice, it is likely that communities will resist the intervention. Conversely, traditionally circumcising communities that perceive infant MC, which allows women (mothers) to see and nurse the circumcision wound as taboo [39], regard the intervention as a serious cultural invasion.

If targets for infant circumcision are to be reached, demand creation initiatives need to change community norms related to infant MC. Beliefs about circumcision are deeply-entrenched and it is likely that demand creation will be a gradual and ongoing process rather than a once-off event. Specifically, demand creation for infant MC for HIV prevention needs to address the wider community and not just mothers and fathers of infant boys since circumcision may have far-reaching social implications for the child in later life. In addition to targeting multiple generations, campaigns should engage key traditional and religious leaders in efforts to mobilise a wider understanding and acceptance of circumcision for HIV prevention [39].

A major strength of this study is that it is the first systematically conducted thematic synthesis to explore parental reasons for non-adoption of infant MC for HIV prevention in sub-Saharan Africa. Given that IMC has been identified as a key HIV prevention intervention for sustaining the prevention gains anticipated through adult voluntary medical MC across sub-Saharan Africa, study findings and resultant recommendations are likely to have broad implications for IMC roll out across the region. Additionally, this study fulfils most of the steps for reporting synthesis of qualitative research as recommended by the ENTREQ statement [22], a valuable and practical resource and reference tool.

Assessing quality of selected studies was difficult. Selected studies were assessed using an adaptation of previously derived (and accepted) quality criteria for assessing validity of qualitative research. The process was challenging and time-consuming; largely because study methods were poorly described or unsystematic. Reviewers then had to try and piece together, from inadequate descriptions, what methods and procedures were used and why [26]. Indeed, it has been suggested that one of the possible by-products of undertaking more qualitative research syntheses may be an improvement in the quality of the reporting of qualitative research [26].

A potential limitation of the study is that we used our own perceptions, as opposed to frequency counts, to determine the weight of the 24 issues (codes) that were identified through line-by-line coding. To some extent, a code’s significance is determined by the number of times it features in a research paper. However, reliance on frequency counts to determine the significance of qualitative findings has inherent shortcomings; the frequency with which an issue is mentioned by research participants may not necessarily be a reflection of its significance but rather, the ease with which it can be mentioned. Also, it could be a result of participants’ conscious efforts to downplay certain issues whilst overstating others. Moreover, presentation of qualitative research is often influenced by the approach taken by authors in conducting and reporting that work; for example authors may sample participants to gain information about specific beliefs or practices or choose to put more emphasis on interesting, novel or unusual findings as opposed to commonly-occurring or previously recognised ones. Additionally, determining the relative weight of the issues identified would always be a subjective exercise and potentially subject to bias (although as stated earlier these biases can be minimised by using at least two experienced researchers to independently code/weight data).

In conclusion, using thematic synthesis, this study identified five key barriers to infant MC uptake (lack of information, fear of harm, cultural/traditional beliefs, concerns about cost and need to respect a child’s autonomy) which were later condensed into just two (poor knowledge and social constructs). Barriers such as knowledge will be relatively easy to overcome but more culturally entrenched beliefs will take time and a layered, community-level approach to change.

## Electronic supplementary material

Below is the link to the electronic supplementary material.

Supplementary material 1 (DOCX 21 kb)

Supplementary material 2 (DOCX 11 kb)
