# Supplementary material for: Factors Associated with Parental Non-Adoption of Infant Male Circumcision for HIV Prevention in Sub-Saharan Africa: A Systematic Review and Thematic Synthesis
Source: AIDS Behav. 2014 Jul 1;18(9):1776–84. doi: 10.1007/s10461-014-0835-7 (PMC4125745; doi:10.1007/s10461-014-0835-7)
Supplement: Supplementary file 1 — Supplementary material 1 (DOCX 21 kb) [file 10461_2014_835_MOESM1_ESM.docx]

**Assessment of quality of included studies**

| **Author/country/date** | **Bailey et al. [37]**  Kenya  **2002** | **Rain-Taljaard et al. [43]**  South Africa  **2003** | **Ngalande et al. [42]**  Malawi  **2006** |
| --- | --- | --- | --- |
| **Paper reports findings from qualitative methodology** | Yes | Yes – Mixed methods | Yes |
| **Clear statement on aims/objectives** | Yes – to assess acceptability and feasibility of MC as an intervention to reduce HIV and STDs | Yes – to assess acceptability of MC as a tool for HIV prevention | Yes – to assess factors to be addressed to enhance MC acceptability should RCTs show that MC prevents HIV |
| **Sampling strategy explained and appropriate** | Yes – FGD participants were selected to represent a range of ages and groups with varying risk profiles | Yes – FGD participants were selected to represent four housing types | Yes – FGDs were conducted in four representative districts of rural/urban Malawi, with various ethnic groups |
| **Data collection methods mentioned/described and appropriate** | Yes – FGDs and semi-structured interviews | Yes – Mixed methods. FGDs and in-depth interviews in combination with two cross-sectional studies | Yes – FGDs |
| **Mention of ethical considerations** | Yes – participants were informed of their right to refuse to answer any question or to withdraw their participation at any time | Yes – participants gave written informed consent | Yes – participants gave verbal informed consent |
| **Theoretical approach mentioned/described** | Not specifically mentioned – used codes from discussion guides and emerging themes | Not specifically mentioned – data were analysed using emerging themes | Not specifically mentioned – used codes from discussion guides, previous studies and emerging themes |
| **Analysis adequately described** | Yes | Yes | Yes |
| **Analysis done by more than one person to minimise subjectivity** | Not discussed | Not discussed | Yes |
| **Results can be linked back to study objectives** | Yes | Yes | Yes |
| **Sufficient data presented to support the results (including quotes)** | Yes – quotes included | Yes – no quotes | Yes – quotes included |
| **Discussion and conclusions adequately supported by the data** | Yes | Yes | Yes |
| **Quality rating** | Good | Fair | Good |

| **Author/country/date** | **Lukobo & Bailey [38]**  Zambia  **2007** | **Mwanga et al. [41]**  Tanzania  **2011** | **Albert et al. [36]**  Uganda  **2011** |
| --- | --- | --- | --- |
| **Paper reports findings from qualitative methodology** | Yes | Yes | Yes – Mixed methods |
| **Clear statement on aims/objectives** | Yes – to assess knowledge, attitudes, beliefs and acceptability around MC to reduce HIV | Yes – to understand attitudes towards MC, policy, regulatory environment and health system readiness for MC | Yes – to assess attitudes and opinions around MC to inform policy |
| **Sampling strategy explained and appropriate** | Yes – FGD participants were selected to represent urban and rural areas plus various ethnic groups and circumcision practices profiles | Yes – key informant interviews were purposively sampled | Yes – FGDs participants were assigned to groups based on sex, age and MC status (men) |
| **Data collection methods mentioned/described and appropriate** | Yes – FGDs | Yes – key informant interviews | Yes – Mixed methods. FGDs were conducted alongside household and provider surveys |
| **Mention of ethical considerations** | Yes – participants were informed of their right to refuse to answer any question or to withdraw their participation at any time | Yes – participants gave written informed consent | No |
| **Theoretical approach mentioned/described** | Not specifically mentioned – used codes from discussion guides, previous study and emerging themes | Yes – thematic coding | No |
| **Analysis adequately described** | Yes | Yes | No |
| **Analysis done by more than one person to minimise subjectivity** | No – just by one person | Yes | Not discussed |
| **Results can be linked back to study objectives** | Yes | Yes | Yes |
| **Sufficient data presented to support the results (including quotes)** | Yes – no quotes just summary of findings | Yes – quotes included | Yes – just two quotes included |
| **Discussion and conclusions adequately supported by the data** | Yes | Yes | Yes |
| **Quality rating** | Fair | Good | Fair |

| **Author/country/date** | **Tarimo et al. [44]**  Tanzania  **2012** | **Milford et al. [40]**  South Africa  **2012** | **Waters et al. [45]**  Zambia  **2012** | **Mavhu et al. [39]**  Zimbabwe  **2012** |
| --- | --- | --- | --- | --- |
| **Paper reports findings from qualitative methodology** | Yes | Yes | Yes | Yes |
| **Clear statement on aims/objectives** | Yes – to assess perceptions of MC among police officers | Yes – to explore views and suggestions regarding introduction of MC as an HIV prevention strategy | Yes – to explore the acceptability of circumcising newborn boys | Yes – to explore acceptability of early infant MC for HIV prevention |
| **Sampling strategy explained and appropriate** | Yes – sampling was done according to age, sex and religion | Yes – key informant interviews were purposively sampled | Yes – FGD participants were purposively sampled | Yes – FGD participants and key informants were purposively sampled |
| **Data collection methods mentioned/described and appropriate** | Yes – in-depth interviews | Yes – key informant interviews | Yes – FGDs | Yes – FGDs and key informant interviews |
| **Mention of ethical considerations** | Yes – participants gave written informed consent | Yes | Yes – participants gave written informed consent | Yes – participants gave written informed consent |
| **Theoretical approach mentioned/described** | Yes – content analysis and SEM enhanced data analysis | Not specifically mentioned – coded data using emerging themes | Thematic content analysis | Principles of grounded theory mentioned but not described |
| **Analysis adequately described** | Yes | Yes | Yes | Yes |
| **Analysis done by more than one person to minimise subjectivity** | Yes | Yes | Yes | Yes |
| **Results can be linked back to study objectives** | Yes | Yes | Yes | Yes |
| **Sufficient data presented to support the results (including quotes)** | Yes – quotes included | Yes – quotes included | Yes – quotes included | Yes – quotes included |
| **Discussion and conclusions adequately supported by the data** | Yes | Yes | Yes | Yes |
| **Quality rating** | Good | Good | Good | Good |
