# Supplementary material for: Factors Associated with Parental Non-Adoption of Infant Male Circumcision for HIV Prevention in Sub-Saharan Africa: A Systematic Review and Thematic Synthesis
Source: AIDS Behav. 2014 Jul 1;18(9):1776–84. doi: 10.1007/s10461-014-0835-7 (PMC4125745; doi:10.1007/s10461-014-0835-7)
Supplement: Supplementary file 2 — Supplementary material 2 (DOCX 11 kb) [file 10461_2014_835_MOESM2_ESM.docx]

**Medline search strategy**

| 1. exp Circumcision, Male/ |
| --- |
| 2. (male or infant* or neonat* or newborn* or child* or baby or babies or son or sons) adj2  circumcis*.mp |
| 3. 1 or 2 |
| 4. HIV Infections/ |
| 5. (HIV adj3 prevent*) or (HIV adj3 intervention*).mp |
| 6. 4 or 5 |
| 7. (accept* or belie* or barrier* or attitude* or willing* or inten* or view* or perspective* or  perceive* or perception*).mp |
| 8. qualitative research/ |
| 9. (focus group* or interview* or qualitative or finding* or theme*).mp |
| 10. 8 or 9 |
| 11. 3 and 6 and 7 and 10 |

Adj2/3 means the two terms are within two or three words of each other, as the case may be

* is a truncation sign
